# Factors Associated With Coronary Artery Disease Testing Among Patients With New-Onset Heart Failure

**DOI:** 10.1016/j.jacadv.2024.101322

**Published:** 2024-10-07

**Authors:** Siamak Kohan, Janet S. Lee, Huong Q. Nguyen, Ming-Sum Lee, Cheng-Wei Huang

**Affiliations:** a Department of Internal Medicine, Kaiser Permanente Los Angeles Medical Center, Los Angeles, California, USA; b Department of Research and Evaluation, Kaiser Permanente Southern California, Pasadena, California, USA; c Kaiser Permanente Bernard J. Tyson School of Medicine, Pasadena, California, USA; d Department of Cardiology, Kaiser Permanente Los Angeles Medical Center, Los Angeles, California, USA; e Department of Hospital Medicine, Kaiser Permanente Los Angeles Medical Center, Los Angeles, California, USA

**What is the clinical question being addressed?** What are factors associated with coronary artery disease testing in new-onset HFrEF?

**What is the main finding?** Variability in coronary artery disease testing among patients with new-onset HFrEF may result from physician perceptions, patient preferences, and socioeconomic factors.

Patients with new-onset heart failure benefit from coronary artery disease (CAD) testing.^1^ CAD testing may be underutilized with reported rates ranging from 20% to 53%, yet the reasons for this potential care gap have not been thoroughly explored.^2^^,^^3^ A deeper understanding of factors that contribute to testing disparities enables targeted intervention to increase testing rates and improve outcomes. We sought to identify factors associated with CAD testing among patients with new-onset heart failure with reduced ejection fraction (HFrEF).

This retrospective cohort study is derived from a pre-existing cohort including adult patients (≥18 years) admitted for new-onset HFrEF between January 1, 2016, and December 31, 2021, within Kaiser Permanente Southern California (KPSC).^1^ KPSC is an integrated health system with 15 medical centers and provides equal access to care for its members, thereby minimizing system-level influences.

In the pre-existing cohort, we had excluded patients who were non-KPSC members (<1 year), pregnant, had a heart transplant history, left against medical advice, discharged to another acute care setting, had an unknown disposition, or had a do-not-resuscitate (DNR) order. In this study, we reincluded those with a DNR order as a surrogate for patient care preferences and further excluded patients who enrolled in hospice, died, or disenrolled from the health plan within 90 days. The pre-existing cohort was limited to HFrEF, given stronger recommendation for intervention within this population.^4^ CAD testing included noninvasive and invasive testing/interventions during and within 90 days of hospitalization.^1^

Multivariable logistic regression adjusting for demographics, comorbidities, socioeconomic status, and clinical variables was performed to estimate the OR of CAD testing for each factor. Variables were selected a priori based on data availability and the authors’ experience. A sensitivity analysis excluding patients who had recent CAD testing within the year prior to hospitalization up to 90 days was also performed. The study was approved by our institutional review board, and informed consent was waived. Analyses were conducted using SAS 9.4 (SAS Institute Inc).

Among 2,930 patients hospitalized with new-onset HFrEF, the median age was 70 (IQR: 58-80) years, 62% were male, and 42% were White. The median ejection fraction was 22% (IQR: 27%-33%). CAD testing was observed in 1,568 (54%) patients (Table 1).

**Table 1:** Characteristics of Patients With Heart Failure With Reduced Ejection Fraction (HFrEF) by Coronary Artery Disease (CAD) Testing and Adjusted Odds Ratio of CAD Testing Within 90 Days

	Total (N = 2,930)	No Testing (n = 1,362)	Yes Testing^a^ (n = 1,568)	Adjusted OR (95% CI)^b^ (n = 2,898)^d^
Age (y)	70 (58-80)	73 (59-84)	68 (57-76)	
<50	365 (12%)	163 (45%)	202 (55%)	0.80 (0.60-1.06)
50-59	449 (15%)	180 (40%)	269 (60%)	Reference
60-69	621 (21%)	224 (36%)	397 (64%)	1.35 (1.03-1.77)
70-79	725 (25%)	298 (41%)	427 (59%)	1.4 (1.01-1.94)
≥80	770 (26%)	497 (65%)	273 (35%)	0.66 (0.47-0.93)
Sex				
Female	1,128 (38%)	552 (49%)	576 (51%)	0.88 (0.75-1.04)
Male	1802 (62%)	810 (45%)	992 (55%)	Reference
Race and ethnicity				
Asian/Pacific Islander	286 (10%)	128 (45%)	158 (55%)	1.15 (0.87-1.52)
Black	556 (19%)	237 (43%)	319 (57%)	1.16 (0.92-1.46)
Hispanic	827 (28%)	377 (46%)	450 (54%)	1.10 (0.90-1.35)
White	1,226 (42%)	605 (49%)	621 (51%)	Reference
Other/unknown^c^	35 (1%)	15 (43%)	20 (57%)	1.02 (0.50-2.06)
Body mass index (kg/m^2^)^d^	29 (25-34)	28 (24-34)	30 (25-35)	
Underweight	50 (2%)	25 (50%)	25 (50%)	1.31 (0.71-2.43)
Normal	691 (24%)	368 (53%)	323 (47%)	Reference
Overweight	906 (31%)	427 (47%)	479 (53%)	1.13 (0.91-1.40)
Obese	1,278 (44%)	539 (42%)	739 (58%)	1.19 (0.96-1.47)
Insurance type^d^				
Commercial/private	1,208 (41%)	478 (40%)	730 (60%)	Reference
Medicaid/dual	133 (5%)	71 (53%)	62 (47%)	0.71 (0.51-0.98)
Medicare	1,510 (52%)	774 (51%)	736 (49%)	0.82 (0.64-1.05)
Neighborhood Deprivation Index (NDI)^d^				
Q1 (least deprived)	732 (25%)	338 (46%)	394 (54%)	Reference
Q2	730 (25%)	340 (47%)	390 (53%)	0.88 (0.71-1.10)
Q3	727 (25%)	337 (46%)	390 (54%)	0.84 (0.67-1.05)
Q4 (most deprived)	729 (25%)	345 (47%)	384 (53%)	0.82 (0.65-1.03)
Comorbidities^e^				
Number of Elixhauser comorbidities	5.0 (3.0-7.0)	5.0 (4.0-7.0)	5.0 (3.0-6.0)	1.03 (0.98-1.08)
Atrial fibrillation	956 (33%)	506 (53%)	450 (47%)	0.69 (0.58-0.83)
Cancer	1,164 (40%)	592 (51%)	572 (49%)	1.17 (0.89-1.54)
Chronic pulmonary disease	803 (27%)	386 (48%)	417 (52%)	0.88 (0.73-1.06)
Diabetes mellitus	1,304 (45%)	589 (45%)	715 (55%)	1.05 (0.88-1.25)
Hypertension	2,457 (84%)	1,147 (47%)	1,310 (53%)	1.06 (0.85-1.34)
Myocardial infarction	557 (19%)	269 (48%)	288 (52%)	0.92 (0.75-1.14)
Renal failure	280 (10%)	125 (45%)	155 (55%)	0.83 (0.69-1.00)
Valvular disease	870 (30%)	390 (45%)	480 (55%)	1.27 (1.06-1.53)
Frailty Index				
Low risk (<5 points)	1,625 (55%)	690 (42%)	935 (58%)	Reference
Intermediate risk (5-15 points)	1,196 (41%)	606 (51%)	590 (49%)	0.93 (0.77-1.11)
High risk (>15 points)	109 (4%)	66 (61%)	43 (39%)	0.64 (0.41-1.01)
Clinical variables				
Do-not-resuscitate order^d^	366 (12%)	265 (72%)	101 (28%)	0.41 (0.31-0.53)
Elevated troponin^f^	218 (7%)	99 (45%)	119 (55%)	1.24 (0.91-1.69)

Values are median (IQR) and n (%).

a Among those tested, 621 (40%) received invasive testing, 738 (47%) received noninvasive testing, and 209 (13%) received both. Noninvasive testing included exercise and pharmacologic stress testing, coronary computed tomography angiography, and cardiac magnetic resonance imaging. Invasive testing/interventions included coronary angiography, percutaneous coronary intervention, and coronary artery bypass grafting. Intervention codes were included to ensure comprehensive capture, as patients would have undergone CAD testing prior to interventions.

b Adjusted for all variables listed.

c Other/unknown further includes multiracial (n = 5), Native American/Alaskan (n = 12), other (n = 14), and unknown (n = 4).

d Missing data (BMI [5], insurance type [3], NDI [12], and code status [12]) were excluded from the multivariable analysis.

e Presence of corresponding International Classification of Diseases codes within 1 y of discharge.

f Defined by hospitalization troponin I >0.50 ng/mL or high sensitivity troponin I >100 pg/mL (male) or >75 pg/mL (female).

In multivariable logistic regression, patients aged 60 to 69 (OR: 1.35 [95% CI: 1.03-1.77]), reference: ages 50 to 59 years), 70 to 79 (OR: 1.40 [95% CI: 1.01-1.94]), and those with valvular disease (OR: 1.27 [95% CI: 1.06-1.53]) had greater odds of CAD testing. Patients who were ≥80 years (OR: 0.65 [95% CI: 0.46-0.93]), had Medicaid/dual insurance (OR: 0.71 [95% CI: 0.51-0.98]), atrial fibrillation (OR: 0.69 [95% CI: 0.58-0.83]), and a DNR order (OR: 0.41 [95% CI: 0.31-0.54]), lower odds of CAD testing.

In our sensitivity analysis, excluding those with recent CAD testing, similar results were generally observed. Though a less pronounced association with increased odds of CAD testing was seen with patients aged 60 to 69 (OR: 1.28 [95% CI: 0.97-1.70]), 70 to 79 (OR: 1.33 [95% CI: 0.95-1.86]), and a more pronounced association with lower odds of testing was seen with renal disease (OR: 0.80 [95% CI: 0.67-0.97]).

In a diverse cohort of patients with new-onset HFrEF, younger age and valvular disease were associated with higher likelihood of CAD testing, whereas older age, Medicaid/dual insurance, atrial fibrillation, renal disease, and DNR status were associated with lower likelihood. These observations suggest that variability in testing may be explained by providers’ perception of HFrEF etiology, their assessment of risks and benefits, patients’ goals of treatment, and socioeconomic factors.

Patients with HFrEF and valvular disease likely underwent CAD testing for risk assessment and prognostic stratification after valvular intervention. Conversely, HFrEF with atrial fibrillation may be attributed to tachycardia-induced HF rather than CAD. For patients with renal disease, less testing may be due to concerns over worsening kidney dysfunction. Furthermore, CAD testing may not be aligned with patients’ priorities, potentially explaining differences by age and code status.

Despite the perceived risk-benefit, we have previously shown that CAD testing improved outcomes in those with atrial fibrillation and renal disease.^1^ This suggests that not all risk-benefit perceptions are accurate, and targeting these subpopulations to improve testing may be beneficial. Additionally, providers should elucidate goals of care rather than presuming that patients with DNR orders would decline interventions.^5^

Notably, even with equal access to care, lower odds of testing were observed among those with lower socioeconomic status and may highlight persistent challenges within this demographic. Social and/or financial constraints beyond access may have hindered these patients’ ability to attend testing or visits to obtain testing referrals.

Our study has limitations, including generalizability beyond KPSC given access differences. While electronic abstraction may result in misclassification, our study benefited from more granular characteristics including socioeconomic status, frailty, and code status. There may be residual confounding, such as patients’ New York Heart Association class, which we did not have information on. Our sample size remains modest and may not have been powered to detect all differences.

Variability in CAD testing among patients with new-onset HFrEF may result from physician perceptions, patient preferences, and socioeconomic challenges. Strategies to better identify patients likely to benefit from CAD testing and bridge the gap in testing among those with lower socioeconomic status deserve further investigation.

## Funding support and author disclosures

This study was supported by the Care Improvement Research Team (CIRT) of 10.13039/100030924Kaiser Permanente Southern California. The authors have reported that they have no relationships relevant to the contents of this paper to disclose.
